# Network Pharmacology and Experimental Validation to Explore the Mechanism of Qing-Jin-Hua-Tan-Decoction Against Acute Lung Injury

**DOI:** 10.3389/fphar.2022.891889

**Published:** 2022-07-08

**Authors:** Shunli Xiao, Lu Liu, Zhengxiao Sun, Xiaoqian Liu, Jing Xu, Zhongyuan Guo, Xiaojie Yin, Fulong Liao, Jun Xu, Yun You, Tiejun Zhang

**Affiliations:** ^1^ Institute of Chinese Materia Medica, China Academy of Chinese Medical Sciences, Beijing, China; ^2^ College of Pharmacy, Henan University of Chinese Medicine, Zhengzhou, China; ^3^ National and Local United Engineering Laboratory of Modern Preparation and Quality Control Technology of Traditional Chinese Medicine, Tianjin Institute of Pharmaceutical Research, Tianjin, China

**Keywords:** Qing-Jin-Hua-Tan-Decoction, acute lung injury, neutrophil extracellular traps, thrombin, network pharmacology

## Abstract

Qing-Jin-Hua-Tan-Decoction (QJHTD), a classic famous Chinese ancient prescription, has been used for treatment of pulmonary diseases since Ming Dynasty. A total of 22 prototype compounds of QJHTD absorbed into rat blood were chosen as candidates for the pharmacological network analysis and molecular docking. The targets from the intersection of compound target and ALI disease targets were used for GO and KEGG enrichment analyses. Molecular docking was adopted to further verify the interactions between 22 components and the top 20 targets with higher degree values in the component–target–pathway network. *In vitro* experiments were performed to verify the results of network pharmacology using SPR experiments, Western blot experiments, and the PMA-induced neutrophils to produce neutrophil extracellular trap (NET) model. The compound–target–pathway network includes 176 targets and 20 signaling pathways in which the degree of MAPK14, CDK2, EGFR, F2, SRC, and AKT1 is higher than that of other targets and which may be potential disease targets. The biological processes in QJHTD for ALI mainly included protein phosphorylation, response to wounding, response to bacterium, regulation of inflammatory response, and so on. KEGG enrichment analyses revealed multiple signaling pathways, including lipid and atherosclerosis, HIF-1 signaling pathway, renin–angiotensin system, and neutrophil extracellular trap formation. The molecular docking results showed that baicalin, oroxylin A-7-glucuronide, hispidulin-7-O-β-D-glucuronide, wogonoside, baicalein, wogonin, tianshic acid, and mangiferin can be combined with most of the targets, which might be the core components of QJHTD in treatment of ALI. Direct binding ability of baicalein, wogonin, and baicalin to thrombin protein was all micromolar, and their K_D_ values were 11.92 μM, 1.303 μM, and 1.146 μM, respectively, revealed by SPR experiments, and QJHTD could inhibit Src phosphorylation in LPS-activated neutrophils by Western blot experiments. The experimental results of PMA-induced neutrophils to produce NETs indicated that QJHTD could inhibit the production of NETs. This study revealed the active compounds, effective targets, and potential pharmacological mechanisms of QJHTD acting on ALI.

## Introduction

Acute lung injury (ALI) and its most severe form, acute respiratory distress syndrome (ARDS), are still the main causes of acute respiratory failure in critically ill patients, with high morbidity and mortality in the past 20 years (Wood et al., 2020). The pathophysiological processes of ALI are believed to involve epithelial and endothelial dysfunction, excessive accumulation, and activation of immune cells, inflammation, oxidative stress, apoptosis, and activation of clotting pathways (Matthay and Zemans, 2011; Matthay et al., 2012; Nadon and Schmidt, 2014). There is currently no specific and effective treatment for ALI. However, there is growing interest in alternative and natural treatments for ALI (Patel et al., 2018).

Qing-Jin-Hua-Tan-Decoction (QJHTD), a classic ancient prescription, listed in the Catalog of Ancient Classical Formulas (first batch released by State Administration of TCM in 2018), which is constituted by 11 Chinese herbal medicines, namely, *Scutellariae Radix*, *Gardeniae Fructus*, *Fritillariae Thunbergii Bulbus*, *Mori Cortex*, *Trichosanthis Semen Tostum*, *Citri Exocarpium Rubrum*, *Platycodonis Radix*, *Ophiopogonis Radix*, *Anemarrhenae Rhizoma*, *Poria*, *and Glycyrrhizae Radix* et Rhizoma. It was first recorded in the ancient book of *Yixue Tongzhi* written by YE Wen-ling in Ming dynasty for treating pulmonary disease with phlegm-heat obstructing lung syndrome, with the significant functions of clearing heat and moistening the lung, reducing phlegm, and relieving cough (Zhang et al., 2021). Previous studies have shown that the pharmacological effects of QJHTD are mainly focused on relieving cough and removing phlegm (Chen et al., 2016), anti-inflammation (Wu et al., 2019), and regulating immune function (Li and Jiang, 2018) in the treatment of pulmonary diseases. It has been reported that QJHTD inhibits LPS-induced ALI (Zhang, 2021). Nevertheless, its exact mechanism of QJHTD on ALI is still unknown.

Network pharmacology is an emerging discipline developed on the network computer platform integrating systematic biology, multi-pharmacology, and computational biology. It helps reveal the mechanism of action of traditional Chinese medicine (TCM) with multicomponent, multitarget, and multisignaling pathways (Hopkins, 2008; Li and Zhang, 2013). Molecular docking is a computational technology that functions through the interaction and affinity between the receptor and drug micromolecules (Chen et al., 2014; Saikia and Bordoloi, 2019), and this method can quickly and effectively screen out the active ingredients. Surface plasmon resonance (SPR) technology has become one of the important means for analysis of small molecules and target proteins, with remarkable features of providing high-precision results and real-time, label-free measurements (Patching, 2014; Nguyen et al., 2015; Olaru et al., 2015; Prabowo et al., 2018).

**FIGURE 1:**
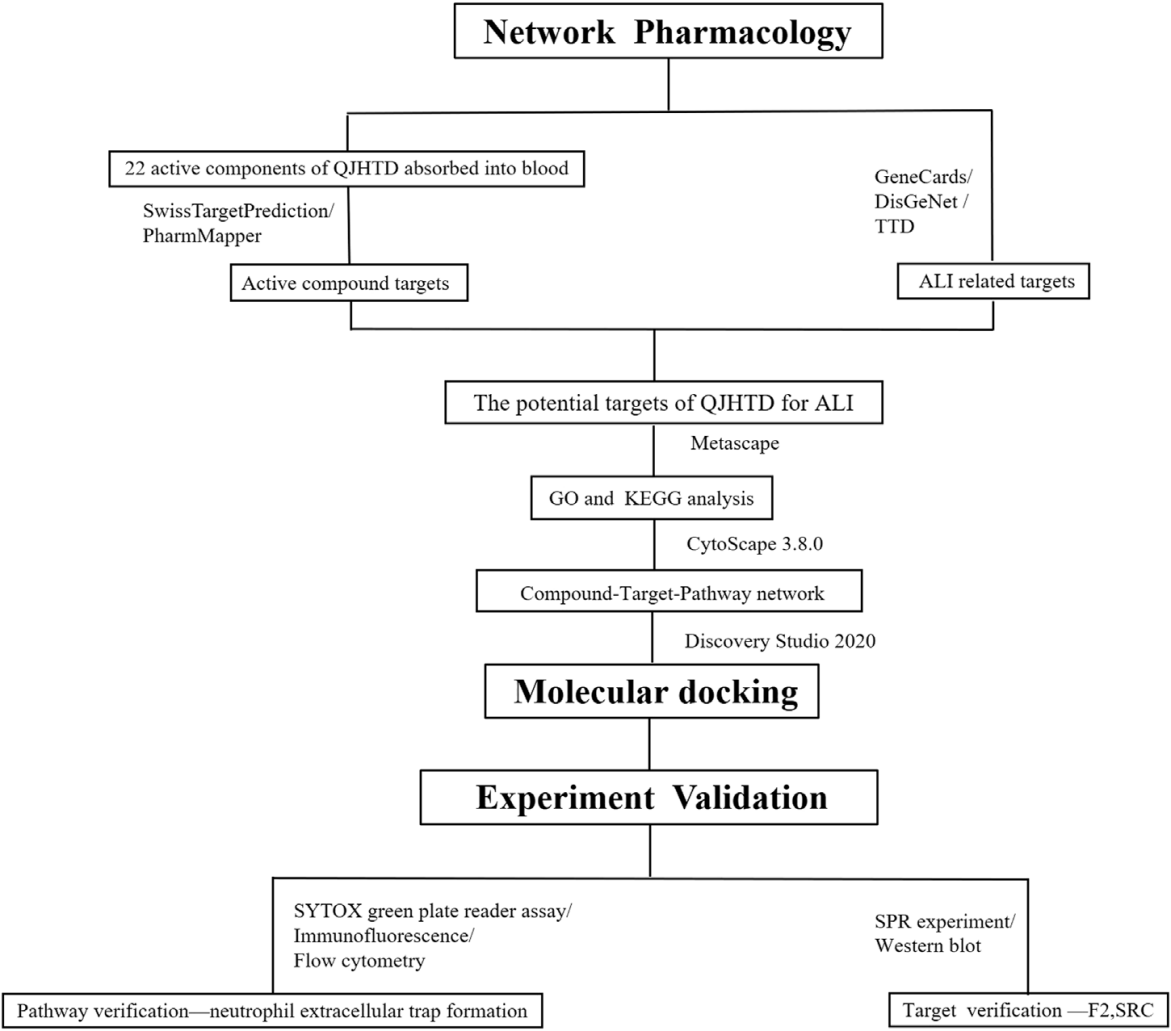
Flow chart of research.

In this research, we adopted the method of network pharmacology combined with molecular docking to screen the possible targets, active components, and signaling pathways of QJHTD against ALI and verified them by SPR technology and cell experiments to further clarify the pharmacological mechanism of QJHTD against ALI. The flow chart of the research is shown in Figure 1. This work provides experimental basis and new mechanisms for the therapy of ALI with QJHTD.

## Materials and Methods

### Identification of Active Components of QJHTD and Their Target Retrieval

**FIGURE 2:**
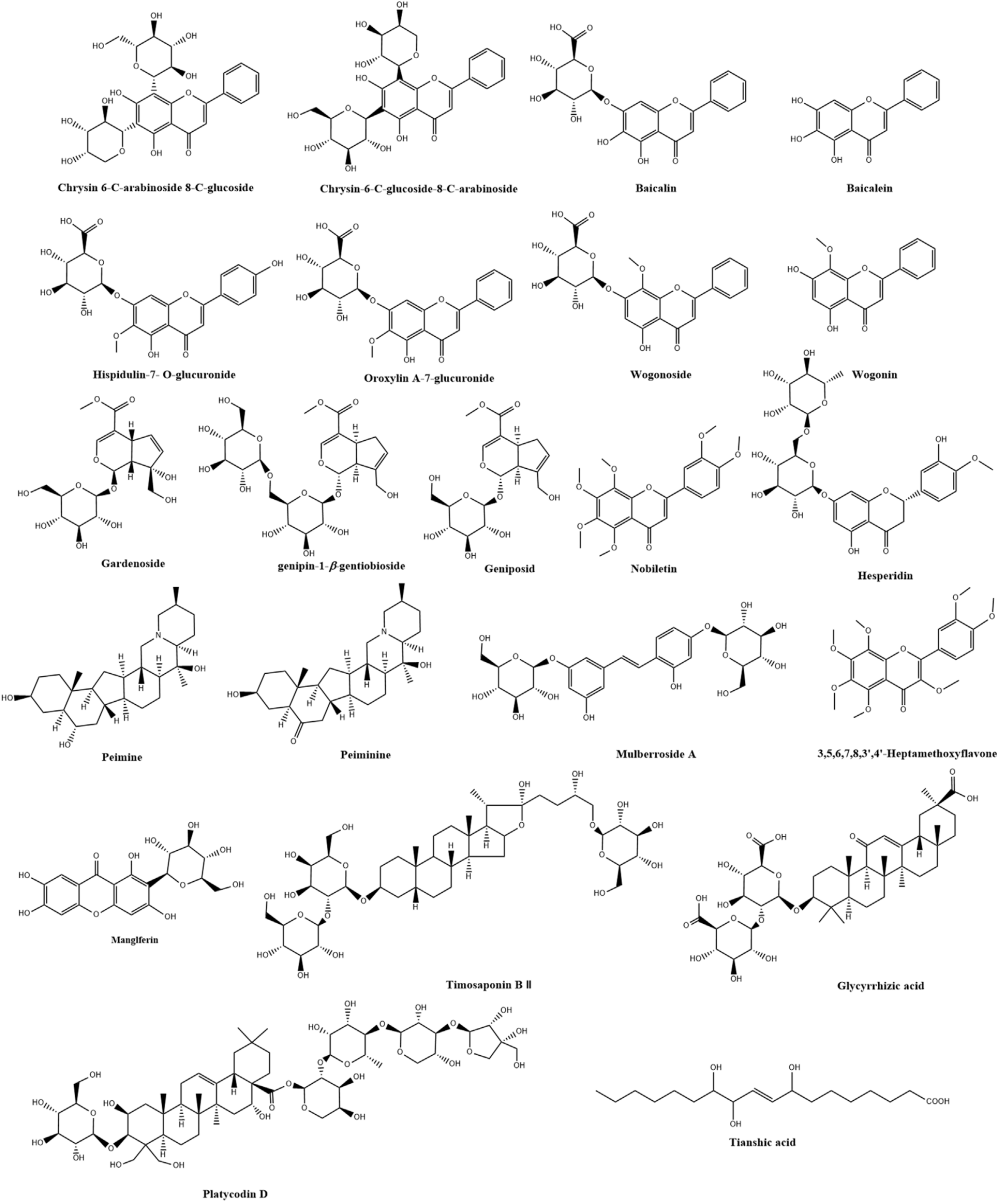
Twenty-two active components of QJHTD and their chemical structures.

A total of 22 prototype components were identified in the rat plasma and are referred to in the previous published literature by the research group (Liu et al., 2022). Chemical characterization analysis and quantitative analysis of QJHTD are shown in Supplementary Material. The chemical structure of 22 active components is shown in Figure 2, and their chemical information is shown in Supplementary Table S1. The SDF format file of the QJHTD components was downloaded from PubChem (https://pubchem.ncbi.nlm.nih.gov/) (Kim, 2016) and uploaded to PharmMapper databases (Liu X. et al., 2010; Wang et al., 2016; Wang et al., 2017) and SwissTargetPrediction. The targets with norm fit ≥0.6 in the output of PharmMapper and convert protein names to official gene symbols (*Homo sapiens*) using UniProt Knowledgebase (http://www.uniprot.org/) (UniProt Consortium, 2018). The potential drug targets predicted by the two databases were selected for further verification.

### Screening Targets of ALI Disease

The information of the therapeutic target was searched by using “acute lung injury” as the keyword. The databases used in this study are GeneCards (https://www.genecards.org/), DisGeNet https://www.disgenet.org/home/), and TTD (http://db.idrblab.net/ttd/). Then, the components and disease overlap proteins are used as candidate targets for the treatment of ALI.

### GO and KEGG Enrichment Analysis

Metascape combines functional enrichment, interactome analysis, gene annotation, and membership search to leverage over 40 independent knowledge bases within one integrated portal (Zhou et al., 2019). The core target proteins of QJHTD for ALI were inputted into Metascape, after which we set *p* < 0.01 to analyze GO and KEGG pathway enrichment. The first 20 KEGG and GO clusters pathway information were screened and included, and the results were saved and visualized by R software.

### Construction of the Compound–Target–Pathway Network

The top 20 clusters obtained from the previous KEGG pathway enrichment analysis correspond to 22 active compounds and the core targets of QJHTD in the treatment ALI and construct the network of “compounds–targets–pathways” using CytoScape 3.8.0 to analyze the network topology parameters of the targets, including degree, betweenness, and closeness.

### Molecular Docking

The virtual docking of key proteins and active components was performed using Discovery Studio 2020 software (College of Pharmacy, Henan University of Chinese Medicine) to study their interaction. For the top 20 targets with a higher degree value in the compound–target–pathway network, the corresponding 3D structure was downloaded in the RSCB PDB database (https://www.rcsb.org/) (Richardson et al., 2021), and 22 component structures were obtained from the PubChem database, and the components were prepared using the “Prepare Ligands” module to obtain the 3D structure. For protein preparation, crystallographic water molecules were removed and then used in the “Prepare Protein” module. Subsequently, CDOCKER was performed for molecular docking (Wu et al., 2003). To enable this mechanism, all default parameters were taken into account, allowing 10 poses to be generated for each ligand. Docking estimation was performed by the CDOCKER energy, which was used to assess the affinity of the proteins and ingredients. 80% of -CDOCKER ENERGY of the target protein and its corresponding prototype ligand was viewed as the threshold, and the components with higher scores were regarded as the active ingredients that interacted with the protein.

## Experimental Validation

### Materials

QJHTD was prepared by Tianjin Pharmaceutical Research Institute Co., Ltd. (Tianjin, China). The preparation process of QJHTD is shown in Supplementary Figure S1. Thrombin (Cat No: HY-114164), dihydrorhodamine 123 (DHR123) (Cat No: HY-101894/CS-7988), and diphenyleneiodonium chloride (DPI) (Cat No: HY-100965) were purchased from MedChemExpress LLC (Monmouth Junction, NJ, United States). Baicalin (CAS No. 21967-41-9), baicalein (CAS No. 491-67-8), and wogonin (CAS No. 632-85-9) were obtained from Shanghai Yuanye Biological Co., Ltd (Shanghai, China), and the purity of all compounds was higher than 98%. The Amino Coupling Kit (Cat No: BR-1000-50), HBS-EP buffer solution (Cat No: BR-1001-88), CM5 Sensor Chip (Cat No: BR-1003-99), and Percoll (Cat No: 17-0891-01) were purchased from GE Healthcare (Braunschweig, Germany). Hoechest 33342 (Cat No: H1399) and SYTOX™ Green Nucleic Acid Stain (Cat No: S7020) were purchased from Invitrogen (Carlsbad, CA, United States). PMA (Cat No: P1585) and LPS (from *Escherichia coli* O111: B4) were obtained from Sigma (St. Louis, MO, United States). Fetal bovine serum (FBS) (Cat No: BR-1003-99) was obtained from Gemini (Woodland, CA, United States). RPMI 1640 medium (Cat No: 10,491), Wright-Giemsa Stain solution (Cat No: G1020), Red Blood Cell Lysis Buffer (Cat No: R1010), Dilution Buffer (Cat No: R1017), Normal Goat Serum (Cat No: SL038), and BCA protein assay kit (Cat No: PC0020) were obtained from Solarbio Life Science (Beijing, China). The ROS detection kit (Cat No: S0033S) and Poly-L-lysine (Cat No: C0313) were obtained from Beyotime (Shanghai, China). Rabbit monoclonal to Src (ab133283), rabbit monoclonal to Src (phospho Y419) (ab185617), rabbit monoclonal to Myeloperoxidase (ab208670), and Alexa Fluor^®^ 488 Goat polyclonal Secondary Antibody to rabbit IgG - H&L (ab150077) were obtained from Abcam (Cambridge, MA, United States). HRP-conjugated goat anti-rabbit IgG antibody (Cat No:bs-40295G-HRP) was obtained from Bioss (Beijing, China). PE anti-rat CD11b/c Antibody (Cat No: B339537) was obtained from BioLegend (San Diego, CA, United States).

### Surface Plasmon Resonance (SPR)

CM5 Sensor Chip was esterified with the crosslinking agents EDC and NHS. The thrombin protein at a concentration of 5 μg/ml in sodium acetate at pH 4.5 was coupled to the surface of the chip, and then the remaining reactive carboxyl on the matrix were blocked using 1 M ethanolamine, at pH 8.5. The compound of baicalin, baicalein, and wogonin were dissolved in DMSO to 10 mM, was diluted with HBS-EP buffer solution to 500 μM, and then diluted successively to 50 μM, 25 μM, 12.5 μM, 6.25 μM, 3.125 μM, 1.5625 μM, 0.7813 μM (baicalin), 12.5 μM, 6.25 μM, 3.125 μM, 1.5625 μM, and 0.7813 μM (baicalein) and 12.5 μM, 6.25 μM, 3.125 μM, 0.7813 μM, and 0.3906 μM (wogonin) using 5% DMSO HBS-EP buffer. The SPR experiment was performed using the Biacore T200 SPR instrument. The injection sample time and velocity were 120s and 20 μl/min, respectively. The protein dissociation time was 300 s.

### Rat Peripheral Blood Neutrophil Isolation

Male Sprague–Dawley rats (220–240 g) were obtained from the Weitonglihua Experimental Animal Technology Co. (Beijing, China) [SCXK 2016–0,006], and housed at 25–28 °C and humidity of 45–55%, with free access to food and drink for 7 days before use.

Rat neutrophils were isolated from the whole blood of healthy rats by gradient centrifugation using Percoll. Briefly, blood was collected from the abdominal aorta of rats and anticoagulated with 109 mM sodium citrate (1:9 blood v/v), and the whole blood was diluted with an equal volume of dilution buffer. Diluted blood was smeared on the interface of the two layers of 82% Percoll and 69% Percoll, and centrifugation was carried out at 710 *g* for 30 min. Neutrophils were collected in the cell layer between the two layers and washed with PBS. Then, red blood cell lysis buffer was added, gently blown for 3–5 min, and incubated at 4°C for 15 min. Centrifugation was carried out at 290 g for 10 min. The supernatant was discarded, and the pellet was washed with PBS and centrifuged again at 250 g for 10 min. The pellet obtained at this point contains the neutrophils. The purity of neutrophil was determined by the flow cytometry and Wright-Giemsa Stain.

### SYTOX Green Plate Reader Assay for NETosis Analysis

Sytox Green dye was used to observe and measure the release of NETs under different conditions (Shi et al., 2019). To quantify the amount of PMA-induced formation of NETs *in vitro*, neutrophils isolated from rat blood (8 × 10^4^ cells/well in 200 µl of medium with 1 μM SytoxGreen) were seeded into 96-well plates. These neutrophils were activated with the media (control), QJHTD at different concentrations (0.125, 0.25, and 0.50 g/L), 50 nM PMA, and 50 nM PMA with QJHTD at different concentrations (0.125, 0.25, and 0.50 g/L), respectively. The plate was placed in a 37°C, 5% CO_2_ incubator for 4 h. Fluorescence was monitored 0 and 4 h using a SpectraMax i3x plate reader (Molecular Devices, San Jose, CA) with excitation at 488 nm and emission at 525 nm. Fluorescence intensity (extracellular DNA) was calculated as (Fluorescence intensity at 4 h) - (Fluorescence intensity at 0 h). The cell status of each group was observed by Axio Observer Z1 (Carl Zeiss AG, Oberkochen, Germany) after the detection with fluorescence microplate.

### NET Induction and Immunofluorescence Staining

Rat peripheral blood neutrophils (1.6 × 10^5^ cells) were seeded on poly-L-lysine-coated coverslips in 48-well plates and cultured for 4 h in RPMI medium containing media (control), 50 nM PMA, or 50 nM PMA with QJHTD at different concentrations (0.125, 0.25, and 0.5 g/L). Subsequently, the cells were fixed with 4% paraformaldehyde for 20 min and permeabilized with 0.2% Triton-X-100 for 20 min. After blocking with 5% normal goat serum for 1 h, it was incubated with rabbit anti-MPO overnight (1:100 dilutions) at 4°C, followed by Alexa Fluor^®^ 488 -conjugated goat antirabbit IgG antibody (1:200 dilutions) for 3 h in the dark. The DNA was counterstained with Hoechst 33342 for 10 min. The images were acquired using Axio Observer Z1 (Carl Zeiss AG, Oberkochen, Germany) and a C11440-42U30 digital camera (Hamamatsu Photonics, Shizuoka, Japan) and processed with BioFlux Montage software (Fluxion Biosciences, Alameda, CA, United States).

### Quantification of ROS Production

The neutrophils were preloaded with DCFH-DA and were diluted at 1:1,000 with PBS. After the extracellular DCFH-DA dye was washed, the cells were resuspended in fresh RPMI medium (8× 10^4^ cells) and were seeded in a 96-well plate. The fluorescence intensity was detected by a SpectraMax i3x plate reader (Molecular Devices, San Jose, CA) at 0, 1, 2, 3, and 4 h, and the excitation wavelength was 488 nm, and the emission wavelength was 525 nm.

Neutrophils were preloaded with 10 μM dihydrorhodamine 123 (DHR123) at 37°C for 20 min. After washing the extracellular DHR123, the cells were resuspended in fresh RPMI medium containing media (control), 50 nM PMA, or 50 nM PMA with QJHTD at different concentrations (0.125, 0.25, and 0.50 g/L) and 50 nM PMA with 20 μM diphenyleneiodonium (DPI) for 1 h. After PBS washing, ROS production was determined using a BD FACSAria II flow cytometer (BD Bioscience, New Jersey, United States).

### Western Blot

According to the network pharmacology and molecular docking results, we selected SRC activity for Western blot verification. The neutrophils were activated using LPS (1 μg/ml), following incubation with or without QJHTD (0.125, 0.25, and 0.5 g/L) at 37°C for 1 h. After 1 h, the cells were added with ice-cold lysis buffer containing protein phosphatase inhibitor mixture, lysed on ice for 30 min, and centrifuged (12,000 rpm, 4°C, 20 min) to obtain the supernatant. Protein concentrations were determined by the BCA protein assay kit. After denaturation by boiling, the proteins were separated by SDS-PAGE and transferred to PVDF membranes. Then, the membranes were blocked with 5% BSA at room temperature for 1.5 h and then incubated with anti-Src (1:1,000 dilution) and anti-Src (phospho Y419) (1:5,000) overnight at 4°C. Subsequently, the membranes were incubated with HRP-linked secondary antibody (1:2,000) at room temperature for 1 h. The results of Western blot were visualized by using an ECL detection system (Syngene, Cambridge, United Kingdom) and analyzed by ImageJ software.

### Statistical Analysis

All data were expressed as the mean ± SD of three independent experiments. The comparison between *multiple* groups was performed by *one*-*way ANOVA* followed by the *LSD* test when the variances were homogeneous or Dunnetts T3 test when the variances were non-homogeneous. All data were analyzed statistically using the SPSS version 23.0 (IBM, Armonk, NY, United States). *p* < 0.05 was considered statistically significant.

## Results

### The Potential Targets of QJHTD for ALI

**FIGURE 3:**
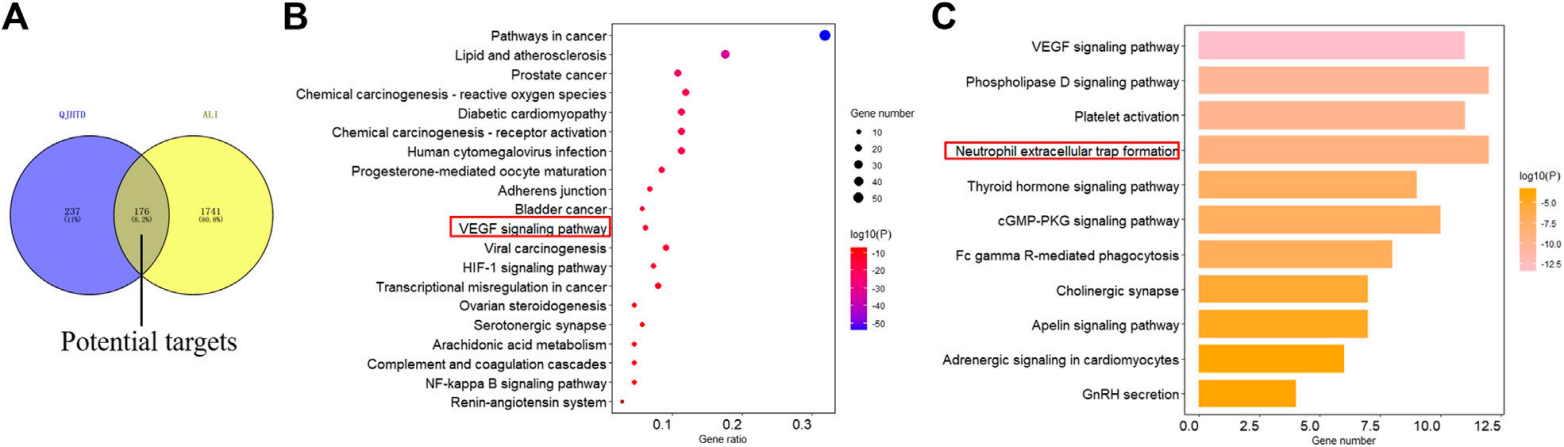
Venn diagram of overlapping targets of QJHTD and ALI; **(A)** top 20 cluster pathways for KEGG enrichment analysis; **(B)** and the 11th clustering pathway expansion diagram **(C)**.

A total of 413 potential QJHTD-related targets were predicted by PharmMapper and SwissTargetPrediction (Supplementary Table S2), and 1,917 disease targets were finally summarized and obtained by removing the duplicate targets by searching the disease databases including GeneCards, DisGeNet, and TTD (Supplementary Table S3). The intersection of the QJHTD-related targets and ALI-related targets has 176 targets (Figure 3A, Supplementary Table S4 **)**, which include AKT1, MAPK1, F2, PPARG, and so on. The 176 targets were considered the potential therapeutic targets of QJHTD for ALI.

### GO and KEGG Pathway Enrichment Analysis of QJHTD for ALI

Metascape was used to analyze the signal pathways of QJHTD-related targets in improving ALI. The results of GO enrichment analysis are shown in Supplementary Figure S4. The biological processes in QJHTD for ALI mainly involved included protein phosphorylation, response to wounding, response to bacterium, regulation of inflammatory response, and so on. Cellular components were related to vesicle lumen, extracellular matrix, platelet alpha granule, and extrinsic component of the plasma membrane. Molecular functions analysis revealed protein serine/threonine/tyrosine kinase activity, endopeptidase activity, nitric-oxide synthase regulator activity, and phosphatase binding. The GO enrichment analysis may be related to the pathogenesis of ALI.

For each given gene list, pathway and process enrichment analysis has been carried out with the following ontology sources: KEGG pathway. All genes in the genome have been used as the enrichment background. Terms with a *p*-value < 0.01, a minimum count of 3, and an enrichment factor >1.5 are collected and grouped into clusters based on their membership similarities. The most statistically significant term within a cluster is chosen to represent the cluster. The first 20 clusters were selected according to their *p*-values to generate the bubble chart for visualization (Figure 3B). It is involved with lipid and atherosclerosis, HIF-1 signaling pathway, transcriptional misregulation in cancer, renin–angiotensin system, VEGF signaling pathway, and so on. From cluster 11, the VEGF signaling pathway expanded, and we can see that it contains VEGF signaling pathway, phospholipase D signaling pathway, platelet activation, neutrophil extracellular trap formation, and so on (Figure 3C). Neutrophils are the host’s first line of defense against microbial infection and play an important role in innate immune response (Nauseef and Borregaard, 2014; Leiding, 2017). Neutrophils survive for a short time in the blood, and they can resist pathogenic microorganisms by phagocytosis, degranulation, and formation of NETs (Kolaczkowska and Kubes, 2013). In recent years, it has been found that in ALI, neutrophil adhesion aggregation and continuous activation lead to an inflammatory cascade, during which a large number of NETs are produced (Saffarzadeh et al., 2012; Luo et al., 2014). Therefore, based on the results of network pharmacology and literature review, we adopted the PMA-induced neutrophil production NET model to verify whether QJHTD treats ALI by inhibiting NETs.

### Compound–Target–Pathway Network

**FIGURE 4:**
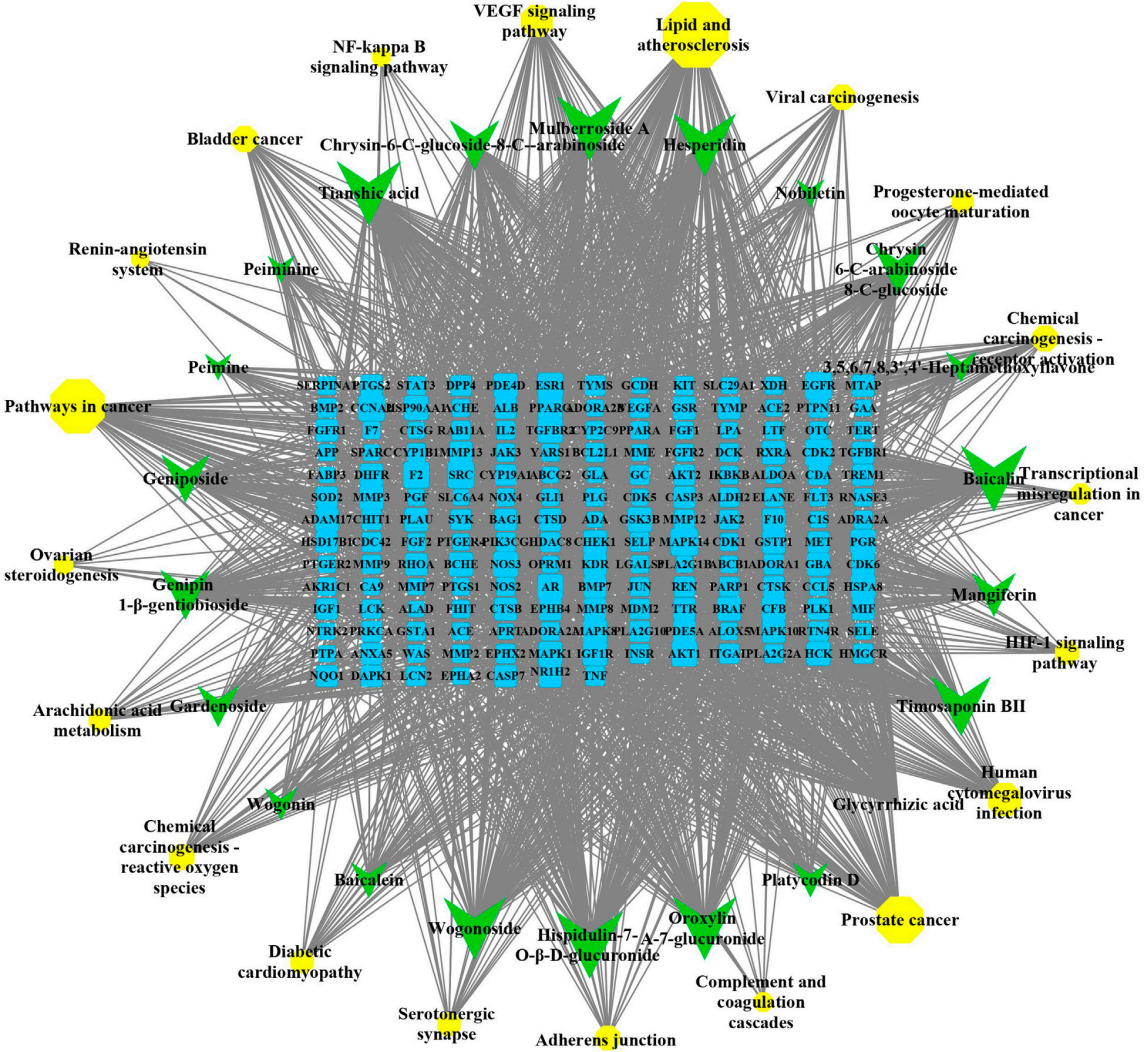
Compound–target–pathway network of QJHTD in the treatment for ALI. The yellow circles, green V shape, and blue square represent the pathway, active components, and targets, respectively.

CytoScape 3.8.0 software was used to construct a component–target–pathway network of QJHTD in treating ALI and was used to calculate and sort the topological parameters (degree) of the nodes in the abovementioned network (Figure 4). The network has 218 nodes, including 22 components, 176 targets, 20 pathways, and 2,044 edges. The larger the node, the greater the degree, and the more nodes connected to it. It was surprised that the degree of MAPK14, CDK2, EGFR, F2, SRC, and AKT1 were higher in the compound–target–pathway network. It was concluded that the 22 active ingredients acted on 176 targets by relevant pathways in lipid and atherosclerosis, renin–angiotensin system, HIF-1 signaling pathway, and so on. Multiple pathways are linked to each other by common targets, indicating the synergistic action of QJHTD in treating ALI.

### Molecular Docking Results

The 22 active components of QJHTD were used as candidate docking components. By analyzing the degree value of the targets in the component–target–pathway network, the first 20 targets (Supplementary Table S5) with higher degree values were selected for molecular docking experiments. The initial compounds of the protein were extracted from the active pockets and redocked and RMSD≤2 evaluated that the docking algorithm could reproduce the receptor-ligand binding mode (Huang et al., 2010).

The original ligands of the selected crystal structures of 20 targets were used to define the active pockets. The original ligands were taken out, and the CDOCKER method was used. Re-dock to the set active pocket, calculate its RMSD and scoring value and its active pocket information, and calculation results are shown in Table 1. It can be seen from the table that the RMSD of the 20 targets are all less than 2 Å, indicating that the selected docking method and parameter settings are reasonable and can be used for further molecular docking research. Molecular docking results in the top 20 target proteins with 22 chemicals and are shown in Supplementary Table S6 and Table 1. The higher -CDOCKER ENERGY is, the more likely the chemical and target are to interact with each other. The results showed that most chemical components had good interaction and binding activities with the targets. Components such as baicalin, oroxylin A-7-glucuronide, hispidulin-7-O-β-D-glucuronide, wogonoside, baicalein, wogonin, tianshic acid, and mangiferin are bound to most of the target proteins, which may be the core components of QJHTD for ALI.

**TABLE 1:** Related information of molecular docking models and molecular docking results of the top 20 targets with 22 active components of QJHTD.

Targets	PDB ID	Radius Å	Active pocket coordinates	RMSD Å	-CDOCKER ENERGY (kcal/mol) of the original ligand	The number of successful components	The number of -CDOCKER ENERGY higher than 80% of the original ligand
MAPK14	1W83	10.040	4.9556, 13.0505, 35.9238	0.6596	41.645	16 (72.23%)	1 (4.55%)
EGFR	5UG9	8.275	−13.8137, 15.0141, −26.6268	1.9704	27.8811	19 (86.36%)	5 (22.73%)
CDK2	1PXK	9.200	12.4244, 45.2819, 24.0306	1.1059	26.7258	17 (77.27%)	6 (27.27%)
SRC	2H8H	9.940	21.0350, 20.1995, 58.5490	1.123	5.4287	20 (90.91%)	13 (59.09%)
CCNA2	4FX3	8.195	−9.7242, 3.4762, 37.1244	0.8398	21.8394	19 (86.36%)	10 (45.45%)
F2	3QWC	7.750	16.8398, −12.7995, 22.4897	0.4607	25.636	19 (86.36%)	3 (13.64%)
AKT1	4EKL	9.495	28.2195, 5.26228, 11.3812	0.702	31.6881	19 (86.36%)	4 (18.18%)
ESR1	5AAV	8.852	31.4186, 12.7432, 11.4012	0.4013	27.683	17 (77.27%)	3 (13.64%)
AR	2PIX	7.500	27.7039, 2.0436, 4.6478	0.2254	6.2037	17 (77.27%)	3 (13.64%)
NOS3	6POV	9.132	−32.2287, −38.6579, −184.9843	0.6297	3.26707	20 (90.91%)	13 (59.09%)
PGR	3HQ5	8.880	−3.0303, −7.6424, 24.3050	0.3023	22.4897	17 (77.27%)	3 (13.64%)
GSK3B	4PTC	7.568	−3.5261, 0.78590, −35.4591	0.4392	35.275	19 (86.36%)	2 (9.09%)
TGFBR2	5E91	10.000	14.9144, −1.0736, 5.5120	1.0171	36.3647	19 (86.36%)	3 (13.64%)
HSPA8	6B1I	8.275	−13.8137, 15.0141, −26.6268	0.7064	58.539	18 (81.82%)	0 (0.00%)
HSP90AA1	6U9A	8.854	3.5789, 9.2703, 26.5332	0.8465	21.1237	20 (90.91%)	5 (22.73%)
IGF1R	1JQH	7.896	28.9641, 58.5570, −8.2719	1.7115	84.4747	22 (100.00%)	0 (0.00%)
PPARG	3OSI	6.610	15.3645, 18.2322, 11.2332	0.3888	32.9185	19 (86.36%)	3 (13.64%)
MMP3	4G9L	7.947	21.6653, 68.3753, 106.167	1.1247	51.0165	20 (90.91%)	1 (4.55%)
PDE4D	1XOQ	11.000	14.0064, 29.3300, 53.1901	0.4731	25.4934	19 (86.36%)	10 (45.45%)
BRAF	3C4C	9.182	0.4785, −2.1111, −19.74544	0.5851	17.0889	18 (81.82%)	6 (27.27%)

**FIGURE 5:**
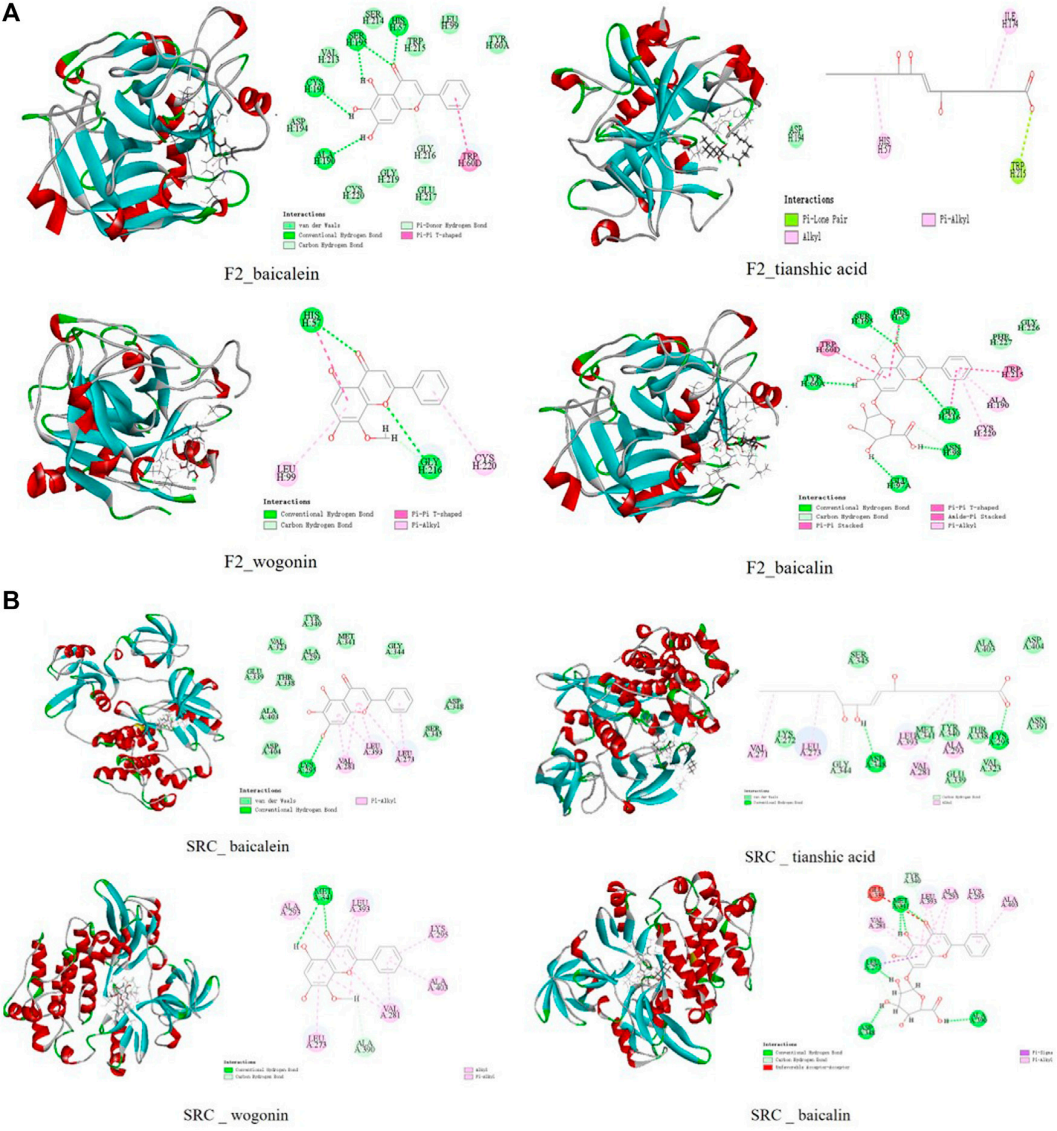
Two- and three-dimensional ligand–protein interactions of baicalein, tianshic acid, wogonin, and baicalin with F2 **(A)** and SRC**(B)**.

Studies have shown that the inflammation of ALI depends on tissue factors and thrombin (Lou et al., 2019). Plasma and lavage fluid thrombin elevated evidently in the animal models of ALI, and thrombin has been found as a key molecule linking coagulation and inflammation (Lou et al., 2019; Arroyo et al., 2021; Kaspi et al., 2021). The results of molecular docking experiments showed that the docking scores of baicalein, wogonin, tianshic acid, and baicalin to F2 (thrombin) reached the effective binding scores. Two- and three-dimensional ligand–protein interactions of baicalein, tianshic acid, wogonin, and baicalin with F2 (PDB ID: 3QWC) are shown in Figure 5A. Both baicalin and wogonin inhibited thrombin-catalyzed fibrin polymerization and platelet functions and inhibited the activities of thrombin (Ku and Bae, 2014; Lee, et al., 2015b). Affinity capillary electrophoresis (ACE) is one of the predominant methods for interaction studies, and baicalein had the greatest affinity with thrombin in comparison with the Kb values of other flavonoid compounds (Li et al., 2018), and it has indicated more OH groups in the A-ring and more thrombin inhibitory activity (Liu L. et al., 2010). Taking into account all these, we thus selected baicalin, wogonin, and baicalein for the experiment of SPR.

The results of molecular docking experiments showed that the docking scores of 13 compounds including baicalein and wogonin to SRC reached the effective binding scores (Supplementary Table S6). Two- and three-dimensional ligand–protein interactions of baicalein, tianshic acid, wogonin, and baicalin with SRC (PDB ID: 2H8H) are shown in Figure 5B. The Src kinase family plays an important role in LPS-induced ALI, and studies have shown that bletinib and resveratrol ameliorates neutrophil inflammation and lung injury *via* inhibition of Src family kinases (Tsai et al., 2019; Kao et al., 2021).

### SPR Experiment Results

**FIGURE 6:**
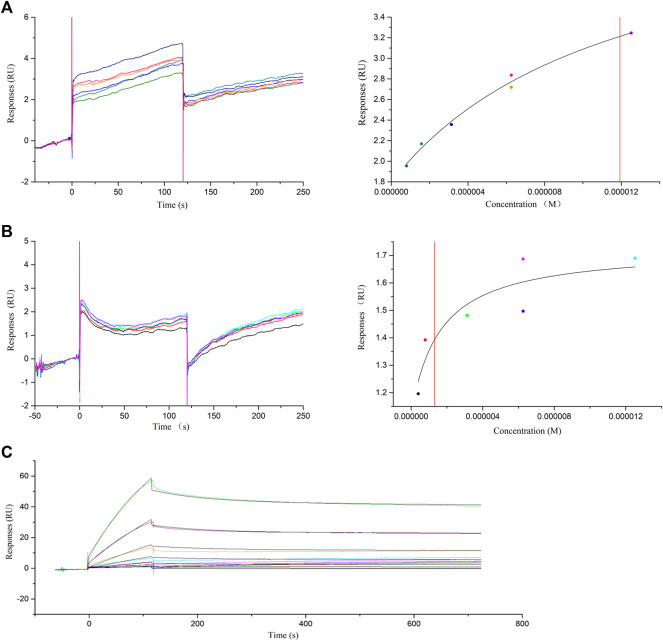
SPR assay of the interaction of baicalein, wogonin, baicalin, and thrombin protein. **(A)** SPR assay of baicalein binding to thrombin (left) and the representative binding curve (right). **(B)** SPR assay of wogonin binding to thrombin (left) and the representative binding curve (right). **(C)** SPR titration curve of baicalin with thrombin protein.

In the SPR experiment, baicalein, wogonin, and baicalin were screened out as *in vitro* validation molecular models, and small-molecule and macromolecular interaction experiments with thrombin protein were performed. The binding affinity (K_D_) describes the strength of the binding between the ligand and the analyzing molecule. K_D_ can be obtained by either “steady-state” or “kinetic” methods. The steady-state method was used in the “fast-up and fast-down” binding mode to obtain affinity, such as baicalein (Figure 6A) and wogonin (Figure 6B). Kinetic analysis K_D_ was obtained from the association rate constant (Ka) and dissociation rate constant (Kd), which was the combined result of the two processes of association and dissociation, such as baicalin (Figure 6C). The results showed that the direct binding ability of baicalein, wogonin, and baicalin to thrombin protein was all micromolar, and their K_D_ values were 11.92 μM , 1.303 μM , and 1.146 μM, respectively (Figure 6). Therefore, it was speculated that baicalein, wogonin, and baicalin bind to the thrombin protein and inhibit its activities and might exert thrombosis prevention in ALI to some extent.

### Rat Peripheral Blood Neutrophil Purity

The purity of neutrophils is >80% with PE Mouse anti-rat CD11b/c antibody was used to label neutrophils (Supplementary Figure S5A). By Wright-Giemsa staining, it was further confirmed that the purity of neutrophils was >80%. At the same time, it could be observed that the neutrophils had complete morphology, the cytoplasm was light red, and the nucleus was purple and lobulated, divided into two–five leaves (Supplementary Figure S5B).

### QJHTD Significantly Inhibited PMA-Induced NETs Formation

**FIGURE 7:**
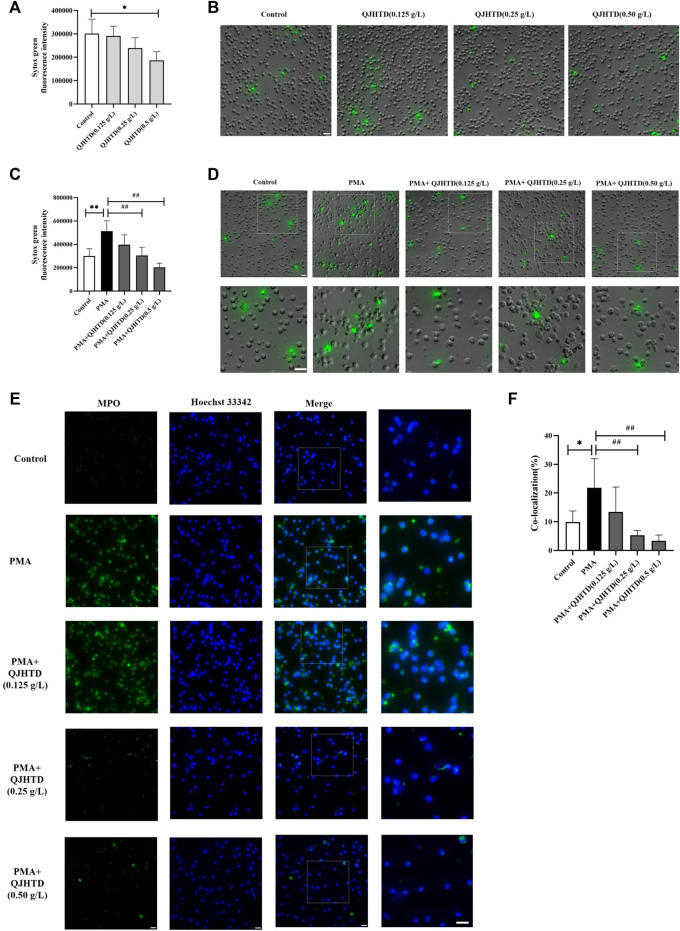
QJHTD inhibited NET formation. **(A)** Effect of different concentrations of QJHTD (0.125, 0.25, and 0.5 g/L) on neutrophils. **(B)** Images of A, scale bar = 20 μm. **(C)** Levels of extracellular DNA released by neutrophils, which were cultured with media, PMA (50 nM), or PMA (50 nM) plus 0.125,0.25, and 0.5 g/L QJHTD. **(D)** Images of C, scale bar = 20 μm. **(E)** Representative images of immunofluorescent staining showed PMA-induced NET formation. Hoechst 33342 (blue), MPO (green), scale bar = 20 μm. **(F)** Quantification data of **(E)**. Data presented as the mean ± SD of three independent experiments (*p < 0.05 and **p < 0.01 compared with control group, ^#^ *p* < 0.05 and ^##^ *p* < 0.01 compared with PMA group).

Phorbol 12-myristate 13-acetate (PMA) is most widely used as an inducer of NETosis. Sytox Green dye is a high-affinity nucleic acid stain that readily cross-damaged cell membranes but does not penetrate the membranes of living cells. At the same time, it could be used to observe and measure the release of NETs (Shi et al., 2019). QJHTD (0.125, 0.25 g/L) showed no significant difference compared with that of the control group (*p* > 0.05), and the immunofluorescence intensity of the QJHTD (0.50 g/L) group was significantly lower than that of the control group (*p* < 0.05), suggesting that the QJHTD group has no cytotoxic effect on neutrophils, and the QJHTD (0.50 g/L) group may play a protective role (Figure 7A). Meanwhile, as shown in Figure 7B, QJHTD (0.125, 0.25, and 0.50 g/L) did not change the morphology of neutrophils.

The immunofluorescence intensity values of the PMA + QJHTD group with different concentrations (0.25 and 0.50 g/L) were significantly decreased compared with those of the PMA group (*p* < 0.01), indicating that QJHTD could inhibit PMA-induced neutrophil releasing NETs (Figure 7C). From Figure 7D, the result was consistent with the fluorescence intensity of each well detected by the fluorescence microplate. It was found that 4 h after PMA stimulation of neutrophils, compared with the control group, the morphology of neutrophils in the PMA group had changed, the cells were flattened, and neutrophils released a large number of green fluorescent filament-like and reticular NETs. Compared with the PMA group, the PMA + QJHTD group with different concentrations (0.25 and 0.50 g/L) did not change the flattened morphology of neutrophils, but the green fluorescent filamentous structures and NETs were significantly reduced, suggesting that QJHTD inhibits the release of NETs from the PMA-stimulated neutrophils.

We measured the release of NETs by detecting the release of MPO-DNA complexes. Immunofluorescent staining of MPO (neutrophil marker) and Hoechst-33342 (nucleic staining) further confirmed the inhibitory effect of QJHTD (Figure 7E). Compared with the control group, neutrophils lost their original structure after PMA stimulation, and their nuclear morphology also showed depolymerization and expansion. There was no significant difference between the PMA + QJHTD group (0.125 g/L) and the PMA group, but the PMA + QJHTD group (0.25 and 0.5 g/L) released significantly less NETs to the extracellular network and filamentous structures (*p* < 0.01) (Figure 7E, Figure 7F). These experiments documented that QJHTD inhibited the formation of PMA-stimulated NETs.

### QJHTD Could Reduce the Generation of ROS During the Formation of NETs

**FIGURE 8:**
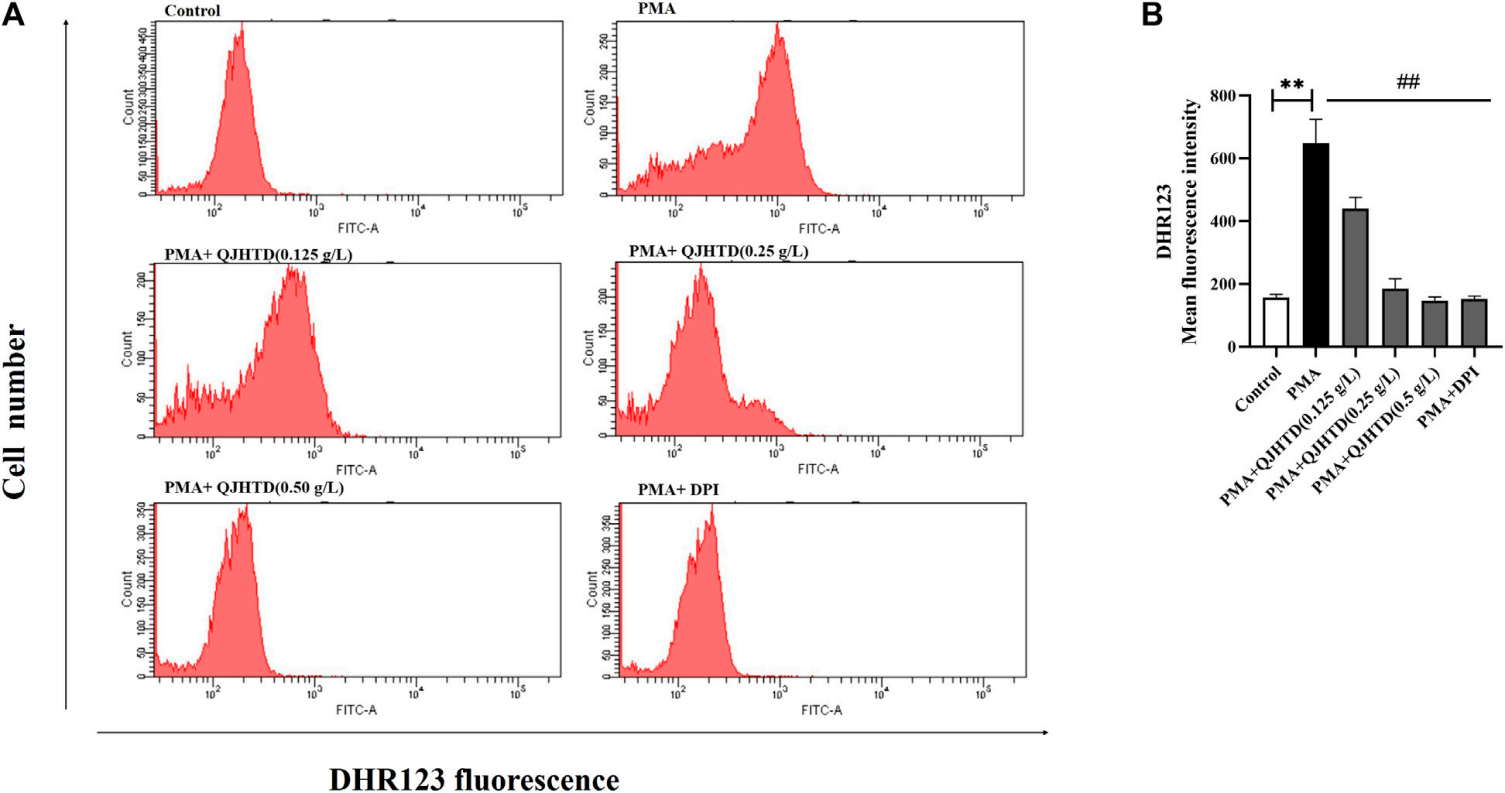
QJHTD could reduce the generation of ROS during the formation of NETs. **(A)** ROS was monitored by flow cytometry using cell-permeable DHR123. **(B)** Quantification data of **(A)** data presented as the mean ± SD of three independent experiments (**p < 0.01 compared with control group, ^##^ *p* < 0.01 compared with PMA group).

NETosis is a multifactorial process, but the detailed molecular mechanisms are not fully understood. The formation of PMA-induced NETs is closely related to ROS generation by nicotinamide adenine dinucleotide phosphate (NADPH) oxidase (Fuchs et al., 2007; De Bont et al., 2018; Fousert et al., 2020). DPI, an NADPH inhibitor, was used as a positive control for the inhibition of ROS production. In the PMA-stimulated neutrophils, the ROS production increased with longer time and the neutrophils were stimulated by PMA (Supplementary Figure S6). As can be seen from Figure 8 and Supplementary Figure S6, QJHTD treatment at concentrations of 0.125, 0.25, and 0.5 g/L significantly inhibited PMA induced ROS production (*p* < 0.01), suggesting that the inhibitory effect of QJHTD on NET formation is mediated by ROS inhibition.

### QJHTD Suppresses Src Phosphorylation in LPS-Activated Neutrophils

**FIGURE 9:**
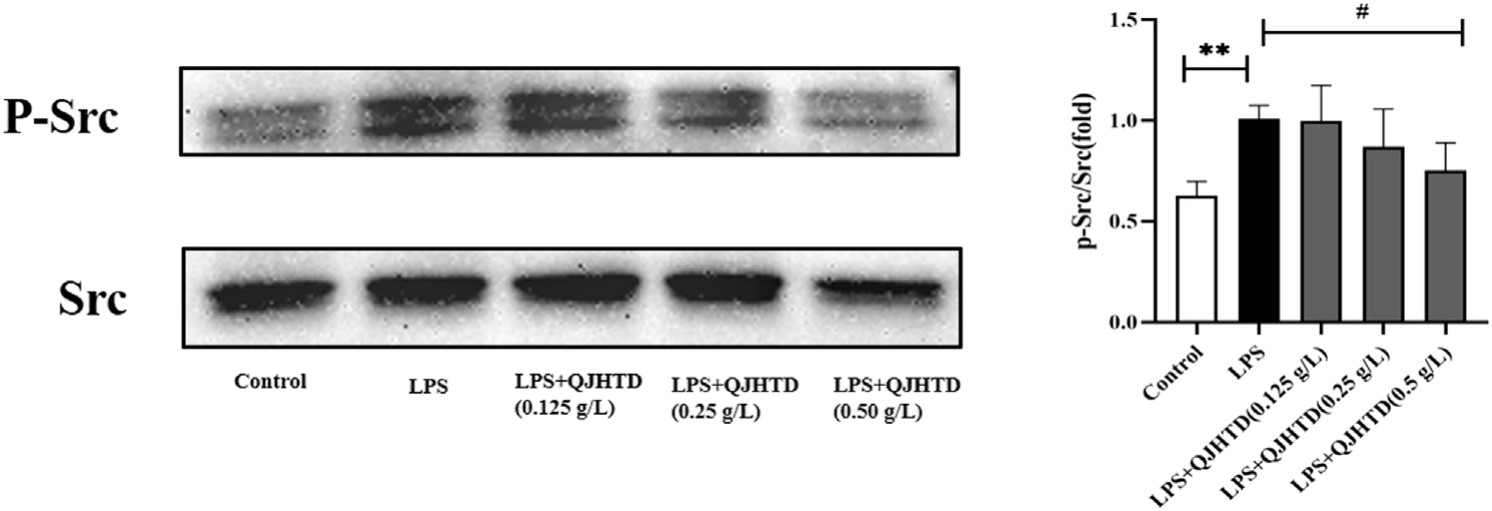
QJHTD suppresses SRC phosphorylation in the LPS-activated neutrophils. Data presented as the mean ± SD of three independent experiments (**p < 0.01 compared with control group, ^#^ *p* < 0.05 compared with LPS group).

The Src kinase family plays an important role in the systemic inflammatory response induced by LPS (Lee et al., 2007; Toumpanakis et al., 2017). Neutrophils have a key role in innate immunity and the development of infections and inflammation, and activation of the Src-dependent Smad3 signaling pathway mediates neutrophil inflammation and oxidative stress (Li et al., 2015). In this study, QJHTD (0.5 g/L) was shown to inhibit the phosphorylation of Src in the LPS-activated neutrophils (Figure 9).

## Discussion

ALI is a diffuse inflammatory response of the lung caused by various internal and external pathogenic factors, which is clinically characterized by respiratory distress and refractory hypoxemia followed by respiratory failure, and with a mortality of approximately 40% (Kallet and Haas, 2003; Matthay and Zemans, 2011). Uncontrolled inflammatory response caused by various immune cells, inflammatory mediators, and cytokines is the main pathophysiological basis of ALI (Tang et al., 2009). Neutrophils play a significant role in the innate immune system. They can quickly migrate to the inflammatory site and digest and destroy the invading pathogenic microorganisms (Nauseef and Borregaard, 2014; Leiding, 2017). Some studies have demonstrated neutrophils exert an important part in the pathogenesis of ALI (Chen et al., 2018; Tsai et al., 2018; Kinnare et al., 2022). Neutrophils play a core role in the initiation, propagation, and resolution of this complex inflammatory environment by migrating to the lungs and performing various proinflammatory functions. These include release of threshing and bactericidal proteins, release of cytokines and ROS, and production of NETs (Potey et al., 2019; Yang et al., 2021).

QJHTD is a classic prescription for the treatment of pulmonary inflammation, and its beneficial effects have been clinically proven. Studies have demonstrated that QJHTD has an obvious effect in the treatment of ALI (Zhang, 2021). Major components of QJHTD such as baicalin (Ding et al., 2016), baicalein (Jiang et al., 2022), wogonin (Takagi et al., 2014), wogonoside (Zhang et al., 2014), geniposide (Xiaofeng et al., 2012), glycyrrhizic acid (Zhao et al., 2016), and platycodin D (Tao et al., 2015) showed good therapeutic effects on ALI. Furthermore, baicalein has been found to inhibit neutrophil respiratory burst activity and its ROS production (Reina et al., 2013). In neutrophils, activated by *N*-Formyl-Met-Leu-Phe (fMLF) or PMA, both baicalein and baicalin effectively blocked the assembly of NADPH oxidase and inhibit the activity of MPO and downregulated the expression of Mac-1, thereby reducing neutrophil adhesion (Shen et al., 2003). Wogonin and wogonoside have also been shown to effectively suppress neutrophil inflammatory activity by inhibiting neutrophil entry into the airways (Takagi et al., 2014) and lung tissue (Zhang et al., 2014; Yeh et al., 2016).

Based on the network pharmacology results, the key targets with higher degree values of the compound–target–pathway network included MAPK14, CDK2, EGFR, F2, SRC, AKT1, and so on. The p38 MAPKs signaling pathway plays an important role in regulating neutrophil activation, especially endotoxin stimulation (Li et al., 2021). In addition, it was demonstrated that MAPK14 was highly expressed in the tissues of ALI mice, and silencing MAPK14 could alleviate ALI symptoms by downregulating inflammatory cytokines (Pan et al., 2019). Inhibition of CDK2 could be used to control neutrophil numbers at the sites of infection or injury, potentially preventing neutrophil-mediated excessive inflammation (Hsu et al., 2019). In lung tissue, EGFR is widely expressed in epithelial cells, and EGFR activation can recruit neutrophils, promoting the secretion of antibacterial peptides and elimination of pathogenic microorganisms (Burgel and Nadel, 2008). EGFR is also involved in regulating the expression of IL-8, thereby promoting the adhesion of neutrophils (Hamilton et al., 2003). Treatment with EGFR inhibitors, such as erlotinib, AG1478, and 451 effectively reduced inflammatory cell infiltration and relieved lung injury in the ALI animal models (Shan et al., 2017; Tao et al., 2019). The Src family consists of non-receptor tyrosine kinases with nine members in total, namely, Src, Fyn, Yes, Yrk, Blk, Fgr, Hck, Lck, and Lyn (Okutani et al., 2006). Several key neutrophil functions are regulated by Src kinases, such as adhesion-dependent degranulation of neutrophils requires the Fgr and Hck (Mócsai et al., 1999). Activation of Src-dependent Smad3 signaling mediates neutrophilic inflammation and oxidative stress in hyperoxia-augmented ventilator-induced lung injury (Li et al., 2015). Studies have shown that bletinib and resveratrol ameliorate neutrophil inflammation and lung injury *via* inhibition of Src family kinases (Tsai et al., 2019; Kao et al., 2021). AKT1 gene deletion can promote neutrophil apoptosis, attenuate neutrophil influx into the lungs of mice, and diminish the expression of proinflammatory factors in bronchoalveolar lavage fluid after intratracheal administration of low-molecular-mass hyaluronan (Zhao et al., 2018). It has also been reported that AKT1 expressed by neutrophils is downregulated during bacterial infection and neutrophil activation, and in the mouse models of ALI and *S. aureus* infection, AKT1 deficiency resulted in severe disease progression with concomitant neutrophil recruitment and enhanced antimicrobial activity, and the AKT1-STAT1 signaling axis may negatively regulate neutrophil recruitment and activation in these ALI mice (Liu et al., 2013). CLLV-1, an AKT inhibitor targeting AKT Cys310, showed potent anti-inflammatory activity in human neutrophils and LPS-induced mouse ALI (Chen et al., 2019).

The top 20 targets with higher degree values of the compound–target–pathway network were selected for molecular docking experiments. The result of molecular docking showed that baicalin, oroxylin A-7-glucuronide, hispidulin-7-O-β-D-glucuronide, wogonoside, baicalein, wogonin, and mangiferin bound with most target proteins. These flavonoids might be the main effective ingredients of QJHTD in the treatment of ALI. The anti-inflammatory mechanism and much more biological activities of baicalein, baicalin, wogonoside, wogonin, and mangiferin have been reported (Chen et al., 2009; Ku and Bae, 2015; Lee et al., 2015a; Qu et al., 2017), such as mangiferin anti-inflammatory by inhibiting the MAPK pathways (Jeong et al., 2014; Suchal et al., 2016; Liu et al., 2019). Wogonin protects against endotoxin-induced ALI *via* reduction of p38 MAPK and JNK phosphorylation (Wei et al., 2017). Components such as baicalin, oroxylin A-7-glucuronide, wogonoside, baicalein, and wogonin are characterized by potential pharmacologically activity in the treatment of virus related to lung inflammation (Li et al., 2011). Meanwhile, both baicalin and wogonin inhibited thrombin-catalyzed fibrin polymerization and platelet functions, prolonged PTT and PT significantly, and inhibited the activities and production of thrombin and FXa (Ku and Bae, 2014; Lee et al., 2015b). The result of molecular docking and SPR experiments suggested that compounds of baicalein, wogonin, and baicalin have a strong affinity with thrombin protein. In the animal models of ALI, plasma and lavage fluid thrombin content elevated evidently (Lou et al., 2019; Arroyo et al., 2021; Kaspi et al., 2021). Neutrophils were shown to be effector cells mediating lung vascular injury after thrombin-induced intravascular coagulation (Malik and Horgan, 1987). Meanwhile, neutrophils were found in large numbers in thrombi within thrombi in injured mice, suggesting that neutrophils are crucial for pathological thrombosis (Brill et al., 2012; Martinod et al., 2013). Moreover, neutrophil binding to endothelial cells was inhibited by ICAM-1 or LFA-1 inhibitors; thus, thrombosis was reduced in mice (Darbousset et al., 2012). In particular, extracellular DNA and histones have been reported to induce thrombin activation *in vitro* (Fuchs et al., 2010; Semeraro et al., 2011), and NETs have been identified in the experimental models of deep-vein thrombosis (De Boer et al., 2013). In *E. coli* induced sepsis, inhibition of NETs attenuates intravascular coagulation and end-organ damage, and blocking NET-induced intravascular coagulation restores microvascular perfusion (McDonald et al., 2017). As mentioned previously, there is a close correlation between thrombin and neutrophils, especially NETs.

GO enrichment analysis of the interactive targets has shown that the biological processes engaged in QJHTD for ALI mainly involved protein phosphorylation, response to wounding, response to bacterium, regulation of inflammatory response, and so on. Molecular functions analysis revealed protein serine/threonine/tyrosine kinase activity, endopeptidase activity, nitric-oxide synthase regulator activity, and phosphatase binding. In terms of cellular components, GO enrichment analysis involved vesicle lumen, extracellular matrix, and platelet alpha granule. More and more studies implicated the potential role of platelet mediators in the pathogenesis and progression of ALI (Looney et al., 2009; Lê et al., 2015; Yasui et al., 2016).

The result of KEGG pathway analysis included neutrophil extracellular trap formation. NETs are a network structure comprising DNA backbone, granule components, histones, and neutrophil elastase and other bactericidal proteins that are released into the extracellular space after neutrophils are stimulated and activated and named this process NETosis (Brinkmann et al., 2004). NETs are a double-edged sword. On the one hand, they can immobilize or trap and kill invading pathogens, exert antimicrobial effects, and facilitate inflammation subsidence, and it is an innate response against pathogen invasion and plays an important role in host defense (Brinkmann, 2018; Petretto et al., 2019; Hilscher and Shah, 2020). However, excessive formation or insufficient clearance can not only directly cause tissue damage but also recruit other proinflammatory cells or proteins, promote the release of inflammatory factors, and further expand inflammatory response (Luo et al., 2014).

For the past few years, NETs have been well-documented in the ALI (Saffarzadeh et al., 2012; Luo et al., 2014; Liu et al., 2016; Gan et al., 2018). Studies have found that NETs are closely related to the damage degree of alveolar epithelial and endothelial cells and the concentration of inflammatory mediators, suggesting that NETs may play an important role in the pathological process of ALI (Saffarzadeh et al., 2012; Luo et al., 2014). Bacteria, viruses, fungi, activated platelets, PMA, and IL-8 can activate neutrophils to generate NETs (Narasaraju et al., 2011; Carestia et al., 2016). We isolated neutrophils from rat peripheral blood, induced neutrophils by PMA, and verified that QJHTD inhibited the formation of NETs by SYTOX Green plate reader assay, fluorescence microscopy, and immunofluorescence stain. These experiments documented that QJHTD inhibited the formation of PMA-stimulated NETs. There are two main pathways for the formation of NETs: pyrolysis NET formation dependent on NADPH oxidase and non-pyrolysis NET formation independent of NADPH oxidase (Chen et al., 2021). PMA activates protein kinase C, which in turn stimulates the production of ROS by activating NADPH oxidase (Fuchs et al., 2007; De Bont et al., 2018; Fousert et al., 2020). DCFH-DA and DHR123 were used as a probe to quantify ROS production, and it was found that QJHTD reduced ROS production during the formation of NETs. Based on the result of *in vitro* experiments, we can speculate that QJHTD plays a crucial role in the treatment of ALI by inhibiting NETs.

## Conclusion

In this study, 22 prototype compounds of QJHTD absorbed into rat blood were combined with the network pharmacology investigation, molecular docking, and experimental validation to elucidate the mechanism of QJHTD against ALI. According to the results, baicalin, oroxylin A-7-glucuronide, hispidulin-7-O-β-D-glucuronide, wogonoside, baicalein, wogonin, tianshic acid, and mangiferin were identified as the vital active compounds, and CDK2, EGFR, AKT1, F2, SRC, and MAPK14 were considered the major targets. SPR experiments also confirmed that baicalein, wogonin, and baicalin have a strong affinity with thrombin protein and might exert thrombosis prevention in ALI to some extent. Western blot experiments demonstrated that QJHTD inhibited Src phosphorylation in LPS-activated neutrophils evidently. Sytox green plate reader assay, fluorescence microscopy, and immunofluorescence stain validated that QJHTD inhibited the formation of PMA-stimulated NETs. This study revealed the active compounds, effective targets, and potential pharmacological mechanisms of QJHTD acting on ALI.

## Data Availability

The original contributions presented in the study are included in the article/Supplementary Material. Further inquiries can be directed to the corresponding authors.
