# Supplementary material for: Network Pharmacology and Experimental Validation to Explore the Mechanism of Qing-Jin-Hua-Tan-Decoction Against Acute Lung Injury
Source: Front Pharmacol. 2022 Jul 8;13:891889. doi: 10.3389/fphar.2022.891889 (PMC9304690; doi:10.3389/fphar.2022.891889)
Supplement: Supplementary file 1 [file DataSheet1.docx]

**Supplementary materials**

**Chemical Characterization Analysis of QJHTD Using UPLC-QTOF-MS/MS**

The UPLC-QTOF-MS/MS analysis was performed on a Waters UPLC coupled with Xevo G2-S QTOF MS system. Chromatographic separation was performed on a Waters BEH C_18_ column (2.1 mm×100 mm, 1.7 μm) at 35 ℃ and flow rate was at 0.4 mL/min. The mobile phases were acetonitrile (A) and 0.1 formic acid in water (B) with a gradient elution program of 5-15 % A at 0-3 min, 15-21 % A at 3-5 min, 21-24 % A at 5-6 min, 24-28 % A at 6-8min, 28-31 % A at 8-10 min, 31-50 % A at 10-14 min, 50-60 % A at 14-15 min,60-95 % A at 15-18 min, 95 % A at 18-19 min. The MS condition was set as follows: capillary voltage, 2.0kV; cone voltage, 40V (ESI+) or (ESI-); source temperature, 120℃; desolvation temperature, 450℃; cone gas flow, 50 L/h; desolvation gas, 600 L/h. The ramp trap collision energy for the high-energy function was set at 25-50 eV. To ensure mass accuracy and reproducibility, the mass spectrometer was calibrated over a range of 100-1600 Da for MS and 50-1600 Da for MS^2^. Leucine-enkephalin (m/z 556.2771 in positive ion mode; m/z 554.2615 in negative ion mode) was used as an external reference for the LockSpray and was infused at a constant flow of 10 μL/min. All MS data were acquired using the MassLynx 4.1 software. The mass spectrum were shown in **Figure S2** and the identified compounds were shown in **Table S1**.

**Quantitative analysis of QJHTD using HPLC**

The HPLC analysis was performed on a Shimadzu LC-20A system. The sample was separated on a Waters Xbridge C_18_ column (4.6 mm×250 mm, 5 μm) at 30 ℃ and flow rate was at 1.0 mL/min. The mobile phases were acetonitrile (A) and 0.1% phosphoric acid in water (B) with a gradient elution program of 5-6 % A at 0-5 min, 6-13.5 % A at 5-25 min, 13.5-21 % A at 25-35 min, 21-23 % A at 35-37 min, 23-24 % A at 37-48 min, 24-26 % A at 48-53 min, 26-28 % A at 53-60 min, 28-40 % A at 60-70 min, 40-60 % A at 70-80 min, 60-80 % A at 80-90 min . The detection wavelength was set at 240 nm. The injection volume for all samples was 10 μL. The HPLC profiles of reference standards and sample were shown in **Figure S3**.


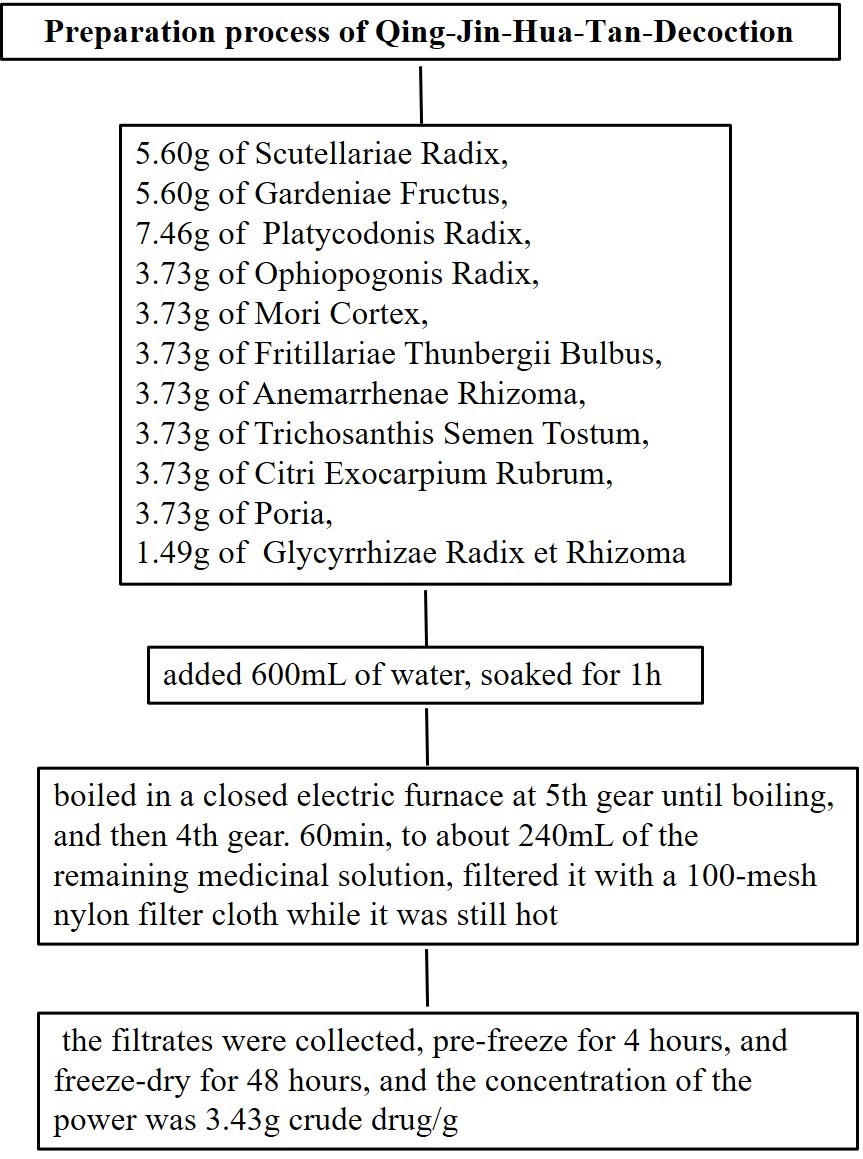


**Figure S1** The preparation process of QJHTD


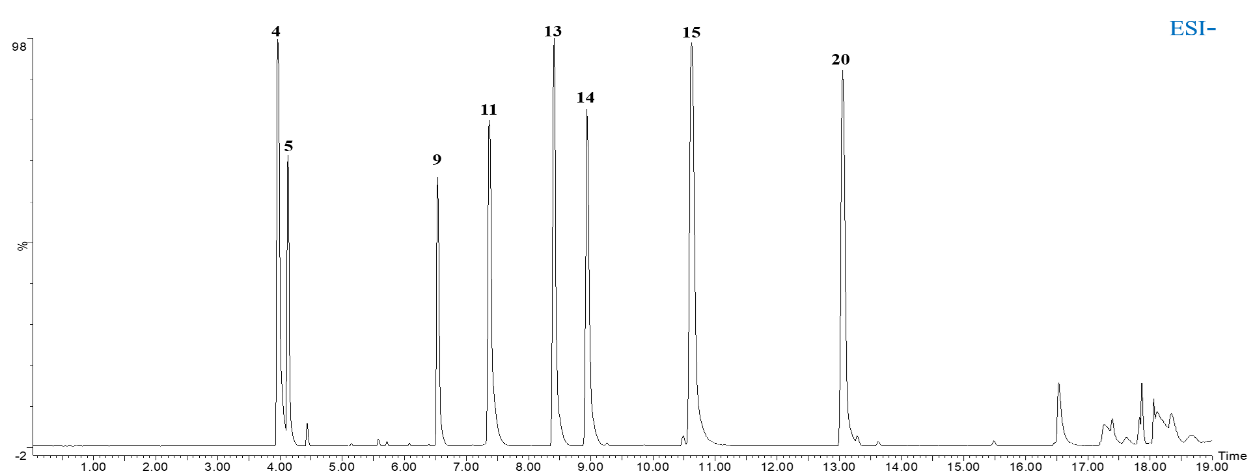


A


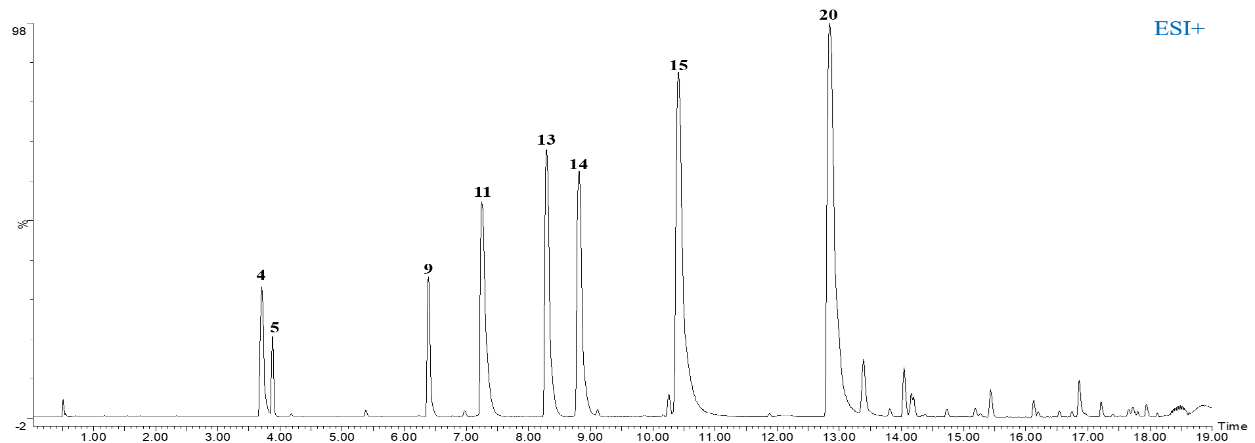


B


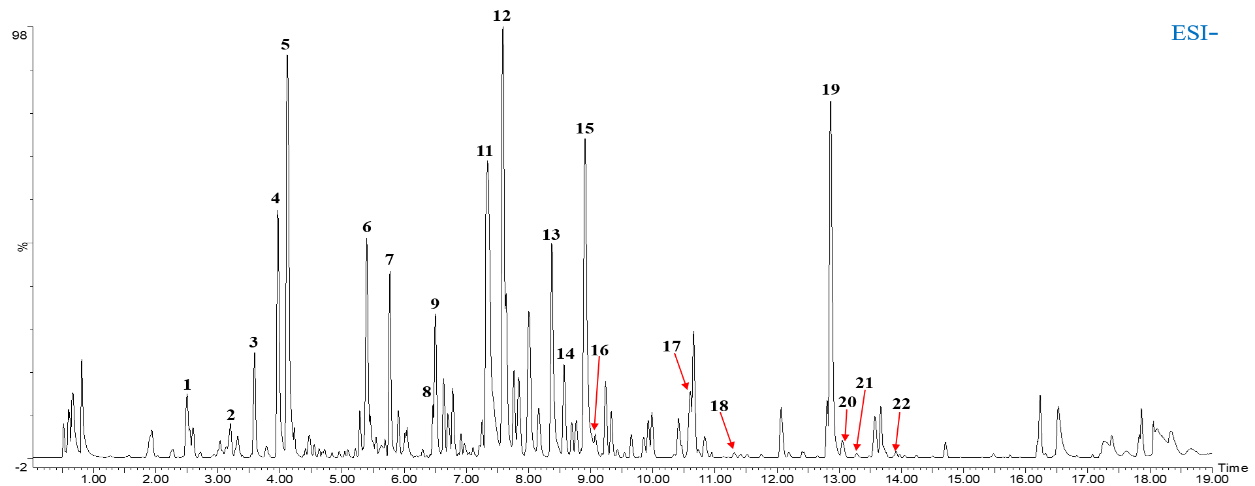


C


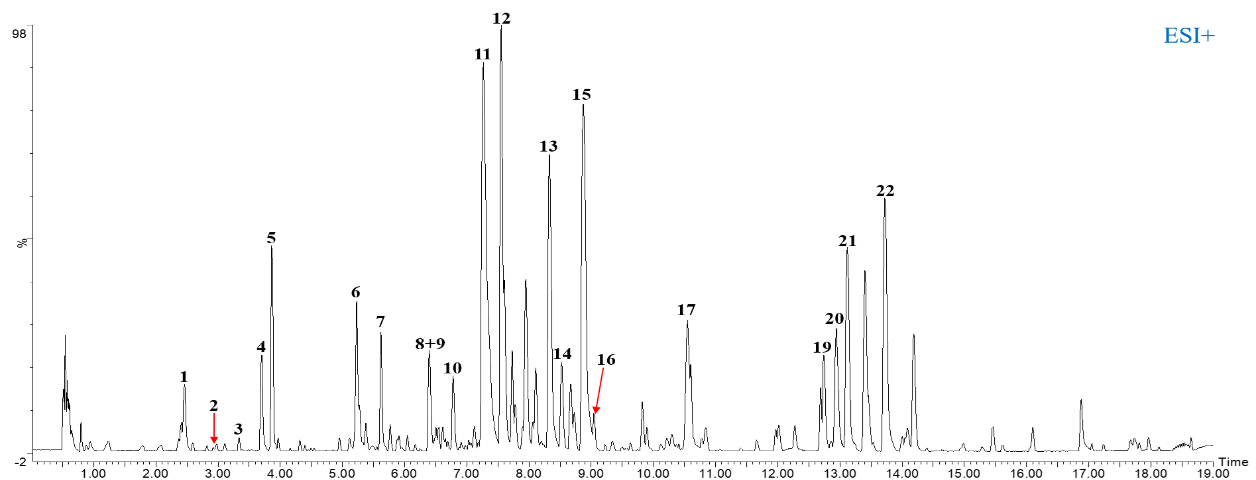


D

**Figure S2** The base peak intensity chromatograms of reference standards (A, B) and QJHTD (C, D).


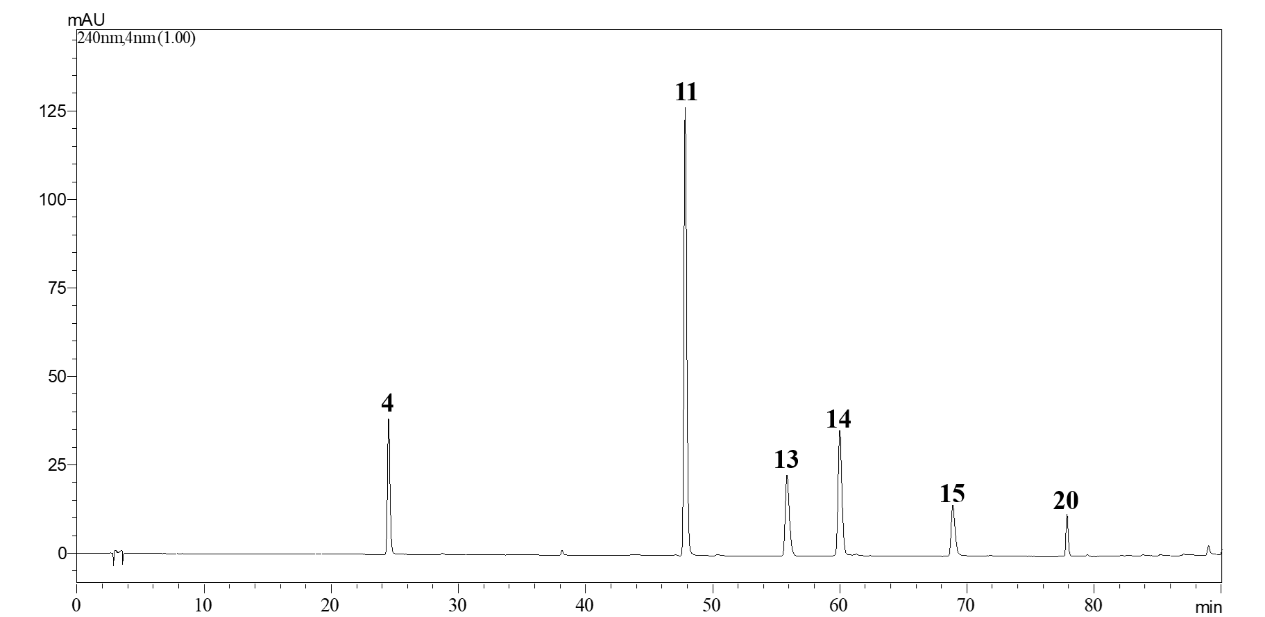


A


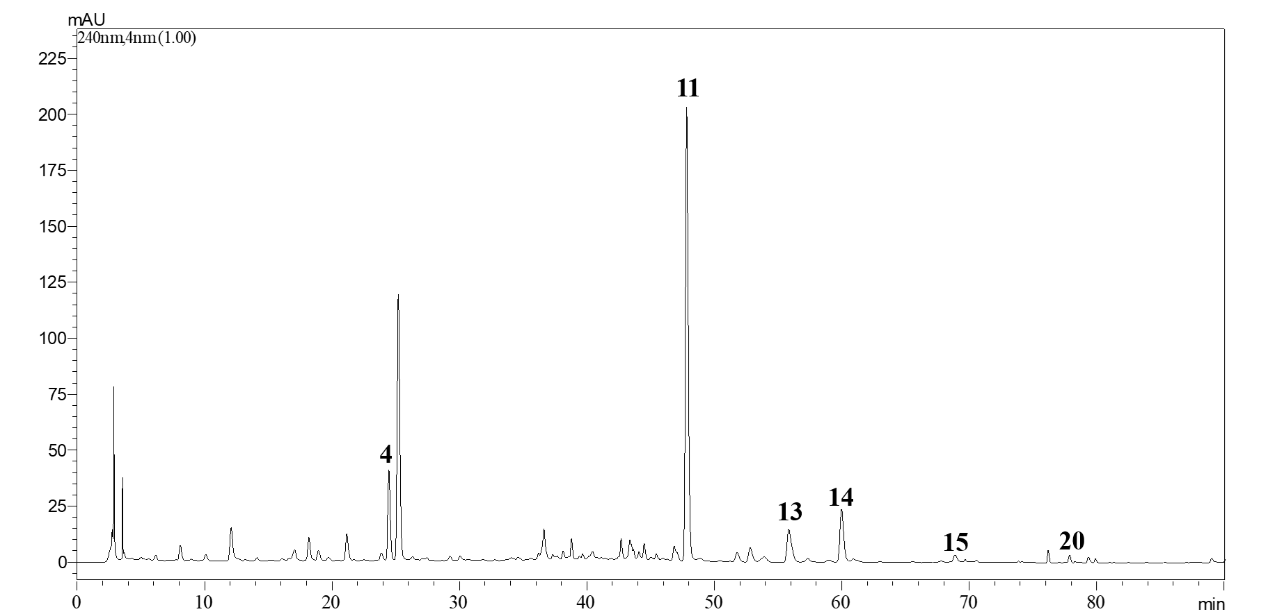


B

**Figure S3** HPLC profiles of reference standards (A) and QJHTD (B). 4, manglferin; 11, baicalin; 13, oroxylin A-7-glucuronide; 14, wogonoside; 15, baicalein, 20, wogonin. The contents of manglferin, baicalin, oroxylin A-7-glucuronide, wogonoside, baicalein, wogonin of QJHTD were 2.44, 40.2, 4.30, 7.97, 0.568, 0.383 mg/g, respectively.


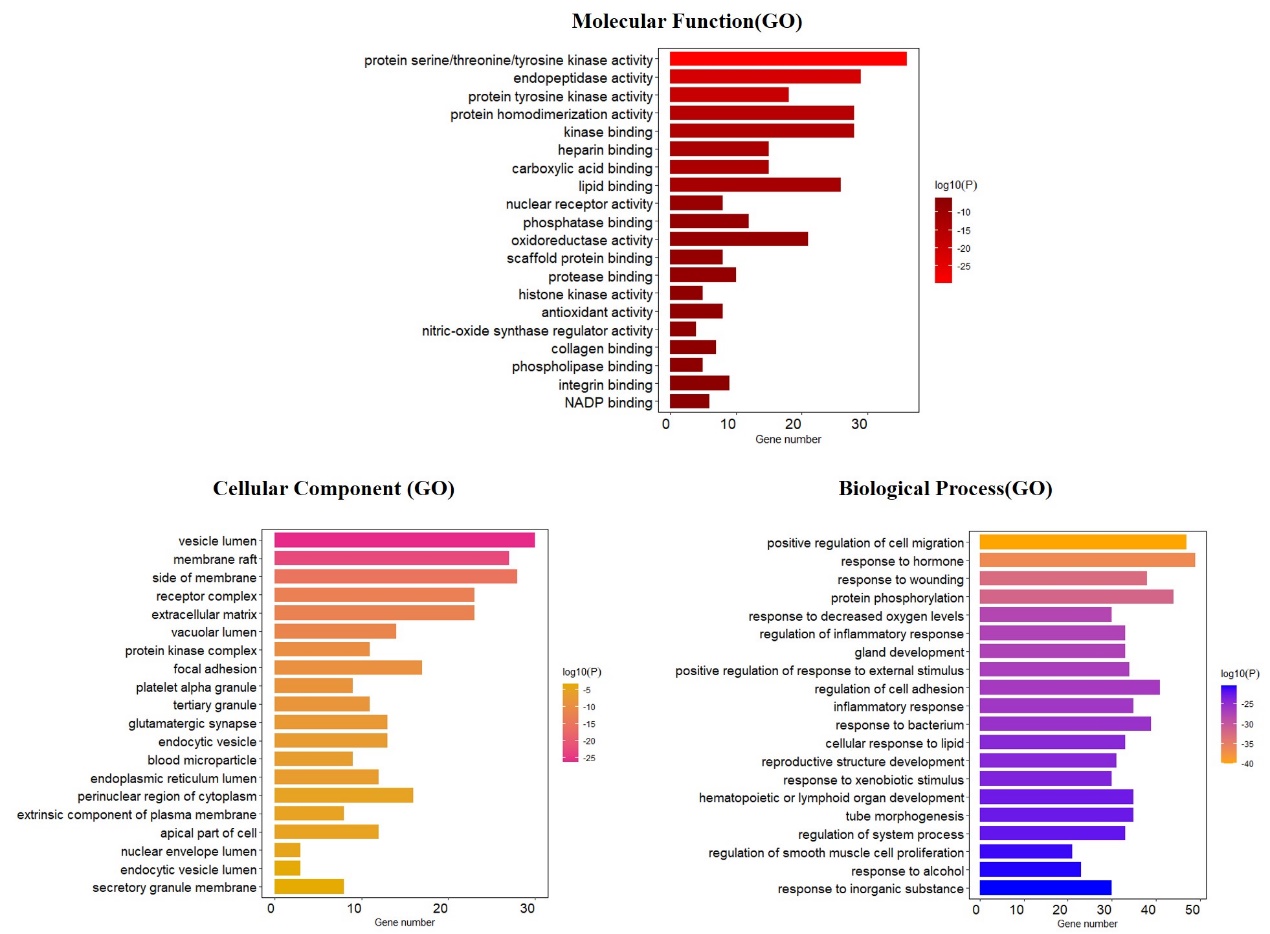


**Figure S4** The top 20 pathways for GO analysis for BP, CC and MF


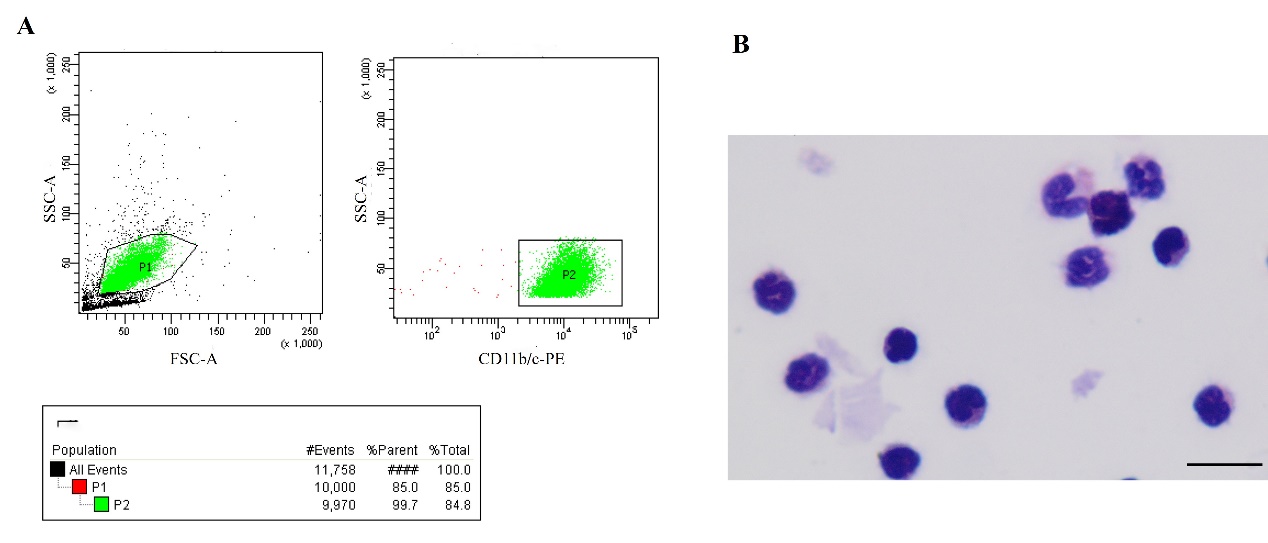


**Figure S5** **Rat peripheral blood neutrophils isolation.**

**(A)** PE Mouse anti-rat CD11b/C antibody tests the purity of neutrophils. **(B)** Wright-Giemsa staining of neutrophils and the scale bar represents 20μm.


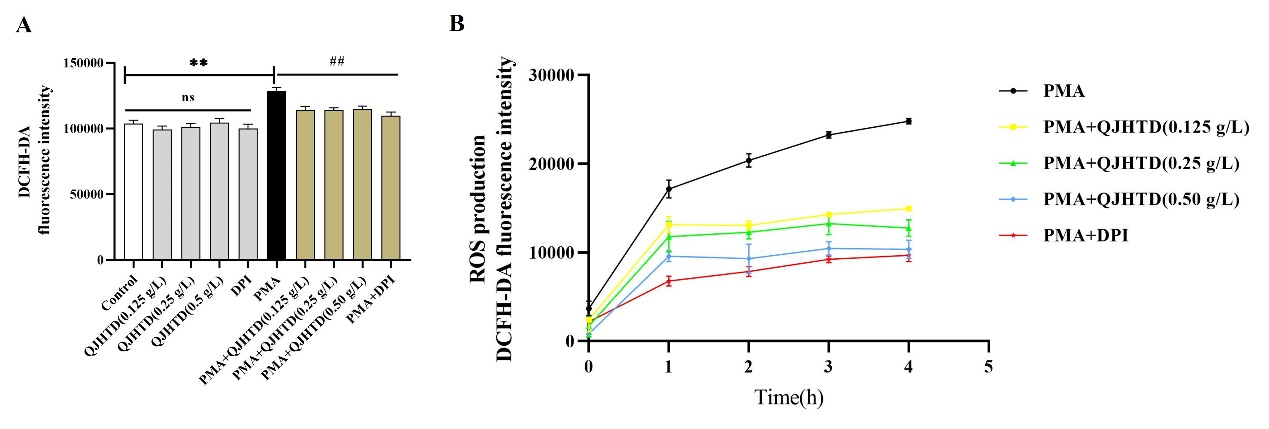


**Figure S6** **The ROS was detected by plate reader using DCFH-DA probe.**

**(A)** The fluorescence intensity of PMA-stimulated neutrophils at the 4h time point. **(B)** The time curve of QJHTD inhibiting ROS production of PMA-stimulated neutrophils. ROS production in PMA group is represented as PMA group fluorescence intensity minus baseline (control group fluorescence intensity）

ROS production in PMA+QJHTD (0.125g/L) group is represented as PMA+QJHTD (0.125g/L) group fluorescence intensity minus baseline (QJHTD 0.125g/L groups fluorescence intensity). ROS production in PMA+QJHTD (0.25g/L) group, ROS production in PMA+QJHTD (0.50g/L) group and ROS production in PMA+DPI group were calculated as above. Data presented as mean ± SD of three independent experiments. (**P < 0.01 compared with control group, ^##^P < 0.01 compared with PMA group, ns indicates not significant)
